# Effects of Burdock tea on recurrence of colonic diverticulitis and diverticular bleeding: An open-labelled randomized clinical trial

**DOI:** 10.1038/s41598-019-43236-0

**Published:** 2019-05-01

**Authors:** Akira Mizuki, Masayuki Tatemichi, Atsushi Nakazawa, Nobuhiro Tsukada, Hiroshi Nagata, Yoshikazu Kinoshita

**Affiliations:** 1 Department of Internal Medicine, Keiyu Hospital, Yokohama, Japan; 2 Department of Preventive Medicine, Tokai University School of Medicine, Isehara, Japan; 3 Department of Internal Medicine, Saiseikai Central Hospital, Tokyo, Japan; 4 Department of Gastroenterology and Hepatology, Shimane University School of Medicine, Izumo, Japan

**Keywords:** Lower gastrointestinal bleeding, Colonic diseases

## Abstract

Colonic diverticular bleeding (CDB) and acute colonic diverticulitis (ACD) show high recurrence rates. The establishment of optimal strategies that prevent the recurrence of CDB and ACD is a major concern among gastroenterologists. This study aimed to assess the efficacy of burdock tea for preventing CDB and ACD recurrences. Newly diagnosed patients with CDB (n = 91) or ACD (n = 70) were randomly assigned into two groups. The experimental group received 1.5 g of burdock tea three times a day, whereas the control group did not receive any treatment. The median (interquartile range) of observation for recurrence of CDB or ACD was 22.0 (14.1) months and 30.3 (18.6), respectively. The burdock tea treatment showed significant preventive effects on recurrence of ACD. A lower ACD recurrence rate (5/47 [10.6%] vs. 14/44 [31.8%]) and longer recurrence-free duration was observed in the burdock tea group (59.3 months [95% CI: 54.0–64.7] vs. 45.1 months [95% CI: 37.1–53.0] by the Kaplan-Meier analysis; p = 0.012 by log rank test) than in the control group, although there was no significant preventive effects on the CDB recurrence. This randomized clinical trial demonstrated that daily intake of burdock tea could be an effective strategy for prevention of ACD recurrence, but not for CDB recurrence.

## Introduction

Colonic diverticular diseases (CDDs) are common in Western and industrialized societies^1^. The prevalence of CDDs is increasing because society is aging^2^. Colonic diverticular diseases place significant economic and clinical burdens on health care systems^3^.

The chief complications of CCDs are colonic diverticular bleeding (CDB) and acute colonic diverticulitis (ACD), and the incident rates of both complications have been increasing. Both CDB and ACD carry a high recurrence rate. Patients who have had experienced an episode of CDB have a 14–38% chance of rebleeding^4,5^. We previously reported a recurrent rate of 34.5% in Japan^6^. As for ACD, 16–42% of patients experience one or more recurrent attacks after nonoperative management of ACD^7–9^. We previously reported a recurrence rate of 36.7% in Japan^10^. Therefore, the establishment of optimal strategies that prevent the recurrence of CDB and ACD is urgently required.

There are three kinds of strategies to prevent the recurrence of CDB and ACD^11^. These are a high fiber diet or supplements, antibiotics, and anti-inflammatory agents. Despite intensive efforts, there are no clear recommendations for prophylactic treatment for the prevention of CCD recurrence. Results on the role of dietary fiber in primary prevention of ACD are particularly conflicting^12–16^. Results of treatment with rifaximin, a broad-spectrum, nonabsorbable antibiotic, are inconclusive^17–20^. The effects of mesalamine, an anti-inflammatory agent (used alone or in combination with antibiotics) are also not clear^21–25^. Raskin *et al*. reported that mesalamine did not prevent recurrent ACD in a large scale, randomized, controlled trial^26^.

Burdock (scientific name: arctium lappa) is a plant which is widely used in Asian medicine as a diuretic antipyretic tea that assists with hypertension, gout, hepatitis, and other inflammatory disorders^27,28^. Pharmacological studies have indicated that burdock roots promote anti-microbial, anti-inflammatory, and free-radical scavenging activity as they contain multiple polyphenols^27–31^. Although the pathogeneses of CDB and ACD are different, burdock tea possibly prevents the recurrence of CDB and ACD given that it is fiber-rich, and contains anti-microbial and anti-inflammatory properties. Thus, we hypothesized that roasted burdock tea could have preventive effects against the recurrence of CDB and ACD. The aim of the present study was to assess the efficacy of burdock tea on preventing the recurrences of CDB and ACD using a random assignment method.

## Materials and Methods

### Ethics

This prospective, randomized, open-labeled, comparative study was approved by the review board of Keiyu Hospital and was performed in accordance with the tenets of the Declaration of Helsinki. This study is registered (UMIN 000022990, 07/02/2016).

### Inclusion and Exclusion Criteria

Newly patients from Keiyu Hospital and Saiseikai Central Hospital, Tokyo were enrolled between April 2012 and December 2016, and tracked until June 2017. Inclusion criteria included patients 1) diagnosed with CDB or uncomplicated ACD and 2) aged between 20 and 85 years.

Exclusion criteria were as follows:

**Study 1**. (1) colon cancer or angiodysplasia, (2) existence of intestinal bowel disease including ulcerative colitis, Crohn’s disease, or Bechet’s disease, (3) pregnancy or possibility of pregnancy, and (4) presence of severe heart failure, liver cirrhosis, chronic hepatic failure, or severe chronic kidney disease.

**Study 2**. (1) colon cancer or acute appendicitis, (2) existence of intestinal bowel disease including ulcerative colitis, Crohn’s disease, or Bechet’s disease, (3) pregnancy or possibility of pregnancy, (4) presence of severe heart failure, liver cirrhosis, chronic hepatic failure, or severe chronic kidney disease, and (5) a history of colonic surgery or colon cancer.

### Protocol

We obtained written, informed consent from each patient before participation in this study. Using simple, computer-based randomization, eligible patients with CDB or ACD were divided into two groups: a tea group (burdock tea [Ajikan, Co., Ltd., Hiroshima, Japan] 1.5 g, t.i.d.) and a control group (no treatment). The treatment with burdock tea was started one month after an initial successful treatment, which was evaluated by ruling out colon cancer using colonoscopy. We had a period of one month to confirm whether the first treatment was successful or not. Especially in ACD, we performed total colonoscopy to rule out colorectal cancers. It was performed more than 2 weeks after the initial treatment to ovoid perforation. During this period, an examination of the whole body was carried out to determine the presence of comorbidity. Therefore, the treatment with burdock tea was started one month after the first treatment.

Patient symptoms were monitored every 12 weeks at outpatient clinics until June 2017. The primary end points were recurrence of CDB and recurrence of ACD. The detailed diagnostic criteria for CDB and ACD are described below. Daily burdock tea compliance was determined by interview at every outpatient visit. In June 2017, all patients were contacted (either at the outpatient clinic or by phone), and information on recurrent CDB or ACD, time of recurrence, lesion site, and type of therapy (conservative or surgery) were obtained.

Information regarding history of CDB and/or ACD, current smoking, alcohol behavior (every day), and usage of anti-coagulant medicines were obtained during interviews.

### Study 1

#### Diagnosis of CDB

The diagnosis of CDB was confirmed by colonoscopy and/or abdominal computed tomography (CT). The diagnosis of CDB was based on the Jensen *et al*. criteria^32^. In brief, patients with a) chief complaint of hematochezia, b) features of CDB on colonoscopy, and c) no bleeding sources in the stomach or duodenum on endoscopy or CT were diagnosed with CDB. An enhanced CT was performed without colonic preparation soon after the patient’s admission. CBD was diagnosed based on following CT findings: extravasation of the contrast medium and no colonic wall thickening. Endoscopic diagnosis of CDB was made upon observation of blood clots in the colon, the presence of diverticula, absence of blood in the terminal ileum, and no other demonstrable cause of bleeding. Small bowel bleeding was excluded based on CT findings such as no extravasation in the small intestine, tumor, or wall thickening of small intestine, as well as by colonoscopy finding such as absence of blood clots in the terminal ileum.

#### Initial treatment procedure

We performed urgent colonoscopy after purge if the patient visited the hospital within 18 hours of hematochezia. In contrast, elective colonoscopy was performed if the patient had a hematochezia episode more than 18 hours before visiting the hospital. This was according to our previous reports^33^. All patients received an oral sulfate purge with polyethylene glycol (PEG) (Niflec, Aginomoto, Tokyo) to rid the colon of clots, stool, and blood. The procedure usually requires 2–4 L of PEG and takes 2–4 h until the colon is sufficiently clean. When active bleeding or signs of hemorrhage were identified on colonoscopy, endoscopic treatment by injection and/or clipping was performed.

Endoscopic injection therapy was based on Jensen’s technique: 1 mL or 2 mL aliquots of epinephrine (1:20,000) were injected into the four quadrants of the bleeding diverticulum^32^. Endoscopic hemostasis was performed by clipping the exposed vessel or erosions (direct method) or the entire diverticular orifice (reefing method) where possible. When bleeding diverticula were not detected by colonoscopy, endoscopic treatment was not performed. Interventional radiology (IVR) and/or surgical intervention was performed when the bleeding site could not be identified by colonoscopy or the bleeding could not be endoscopically controlled.

### Study 2

#### Diagnosis of ACD

Acute colonic diverticulitis was suspected in patients with lower abdominal pain, abdominal tenderness on physical examination, and leukocytosis on laboratory testing. The diagnosis was confirmed by ultrasound (US) and/or CT. The US criteria for the diagnosis of ACD were according to the US grading system (grade I and II): grade I indicated an inflamed diverticulum with/without pericolitis and an abscess of ≤3 cm in diameter; grade II indicated an inflamed diverticulum with an abscess of >3 cm in diameter or a perforation^33,34^. The CT criteria for moderate ACD were localized thickening of the colonic wall (5 mm or more) and inflammation of the pericolic fat. The criteria for severe ACD included abscess, extraluminal air, and/or contrast^35^. All patients who met the inclusion criteria and did not meet the exclusion criteria were enrolled into the study, regardless of whether the ACD was right-sided or left-sided.

#### Initial treatment procedure

Patients with grade I-ACD were treated as outpatients or inpatients. A 10-day treatment protocol was performed for outpatients^33^. This consisted of an oral antibiotic (cefpodoxime proxetil 200 mg, b.i.d. for 10 days) with at least 1500 mL/day of a sports drink (405 kcal, 27 kcal/100 mL) for the first 3 days. Free intake of water was allowed during the first 10 days. If the patient showed improvement by day 4, a clear liquid diet was permitted. If improvements were still evident on day 7, a regular diet was resumed. Where there was no improvement, the patient was hospitalized and administered intravenous antibiotics. After clinical signs of local inflammation had disappeared completely (typically in six to eight weeks), a colonoscopy was performed to confirm the presence of diverticula and rule out colon cancer.

Patients who were judged treatable as outpatients because US and/or CT indicated uncomplicated ACD were treated according to a 10-day treatment protocol. Outpatients with any of the following conditions were excluded: (1) Grade-II ACD, (2) complicated ACD, (3) bacteremia, or (4) any severe comorbidity such as uncontrolled diabetes mellitus, heart failure, renal failure, or end-stage cancer.

In addition, patients who had undergone pre-treatment involving broad-spectrum antibiotics (within 24 hours prior to presentation in the outpatient clinic) and those deemed unable to understand the protocol or manage self-care were also excluded.

Inpatient treatment of ACD varies depending upon whether the patient has complicated or uncomplicated disease. Patients with complicated diverticulitis must undergo treatment specific to their complications. All patients undergo treatment for ACD with intravenous antibiotics, fluids, and pain medications.

### Statistical analysis

Student t tests and a chi-squared test were used to assess the significance of differences between the two groups. Continuous data were expressed as either mean ± standard deviation (SD) or median (interquartile range [IQR]). The CBD-free or ACD-free period was calculated from the date of the patient’s first attack to the date of CBD or ACD recurrence. In patients without recurrence, the CBD-free or ACD-free period was calculated from the date of the patient’s first visit to hospital to the final follow-up date (June 2017). Outcomes were compared between two groups using the Kaplan-Meier method. Statistical significance was determined using the log rank method.

All statistical analyses were performed using IBM-SPSS ver. 24 (IBM-SPSS, Inc., Tokyo, Japan).

## Results

### Patient characteristics

In this study, actual average compliance in CDB and ACD were 97.7% and 96.6%, respectively. Drinking burdock tea three times a day was considered as a good compliance. Of 47 ACD patients, one drank burdock tea once a day and one drank twice a day. Of 37 CDB patients, one drank burdock tea twice a day. Other participants drank burdock tea three times a day, according to the protocol.

### Study 1

**Figure 1:**
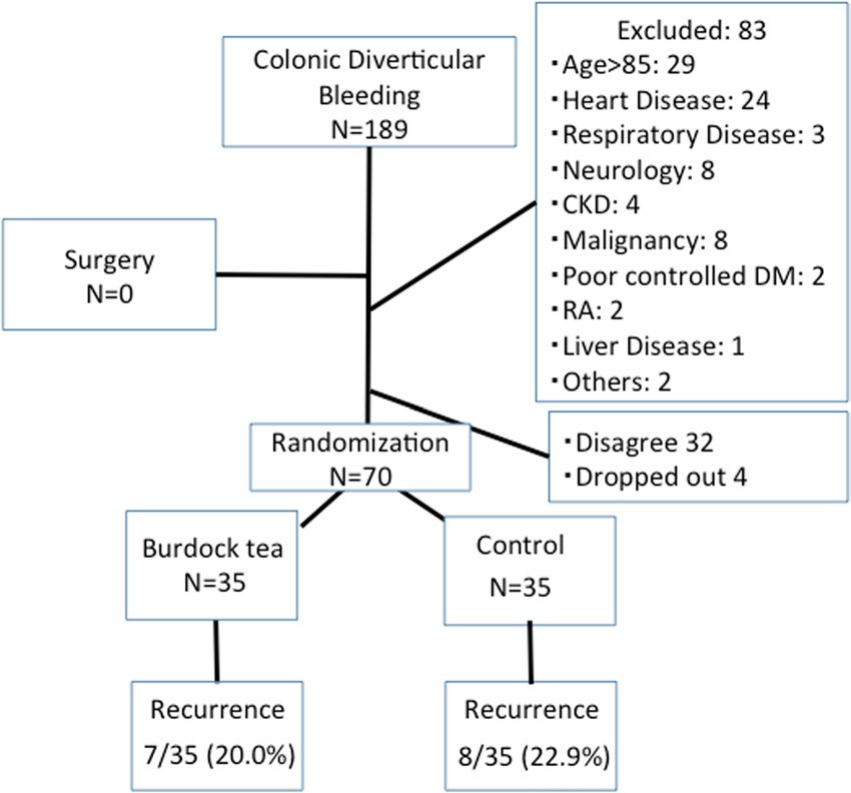
The flow chart of the study population in Study 1.

Seventy patients (52 males, 18 females; mean age [SD]: 66.6 [11.1] years) were initially treatable. Table 1 summarizes the patients’ characteristics. No significant differences were noted between the two groups. A flow chart of the participants’ allocations are shown in Fig. 1.

**Table 1:** Patients Characteristics of Colonic Diverticular Bleeding.

	Budock Tea n = 35	Control n = 35	*p*
Mean (range) age (yr)	66.2 (49–79)	66.9 (41–82)	0.808
Sex (M/F)	26/9	26/9	0.779
BMI (kg/m2)(mean(SD))	24.2 (2.8)	22.7 (5.2)	0.164
History of CDB (%)	9 (25.7)	8 (22.9)	0.780
History Of ACD (%)	6 (17.1)	5 (14.3)	0.743
Drinking (%)	20 (52.6)	18 (51.4)	0.631
Smoking (%)	4 (12.1)	7 (20.0)	0.378
Comorbidities (%)	22 (62.9)	18 (51.4)	0.334
NSAIDs (%)	1 (2.9)	3 (8.6)	0.303
Antiplatelets (%)	4 (11.4)	6 (17.1)	0.495
Anticoagulant (%)	2 (5.7)	4 (11.4)	0.393
Hb (g/dl)(mean(SD))	12.3(2.0)	11.4 (2.8)	0.133
Visible vessels (%)	3 (8.5%)	5 (14.2%)	0.485

ACD: acute colonic diverticulitis, CDB: colonic diverticular bleeding.

### Study 2

Ninety-one patients (47 males, 44 females; mean age [SD]: 51.0 [13.0] years) had been initially and successfully treated. Table 2 summarizes the patients’ characteristics. The number of patients with history of ACD was significantly higher in the burdock tea group than in the control group (p = 0.034). No significant differences were noted between the two groups for any of the other parameters.

**Table 2:** Patients Characteristics of Acute Colonic Diverticulitis.

	Budock Tea n = 47	Control n = 44	*p*
Mean (range) age (yr)	48.0 (24–82)	53.0 (27–79)	0.119
Sex (M/F)	26/21	21/23	0.469
BMI (kg/m2)(mean(SD))	22.8 (4.6)	22.4 (3.4)	0.588
Location of ACD (Right/Left)	31/16	33/11	0.345
History of ACD (%)	18 (38.3)	8 (18.2)	0.034
History of CDB (%)	4 (8.5)	2 (4.5)	0.448
Drinking (%)	25 (53.2)	22 (50.0)	0.761
Smoking (%)	9 (19.1)	15 (34.9)	0.092
Comobidities (%)	20 (42.6)	21 (47.7)	0.62
WBC (/mm3)(mean(SD))	11268.4 (3901.9)	11007 (4004.7)	0.760
CRP (mg/dl)(mean(SD))	7.2 (5.7)	6.1 (3.8)	0.298
Out-patient treatment (%)	23 (48.9%)	15(34.1%)	0.153

ACD: acute colonic diverticulitis, CDB: colonic diverticular bleeding.

### Recurrence

#### Study 1

**Figure 2:**
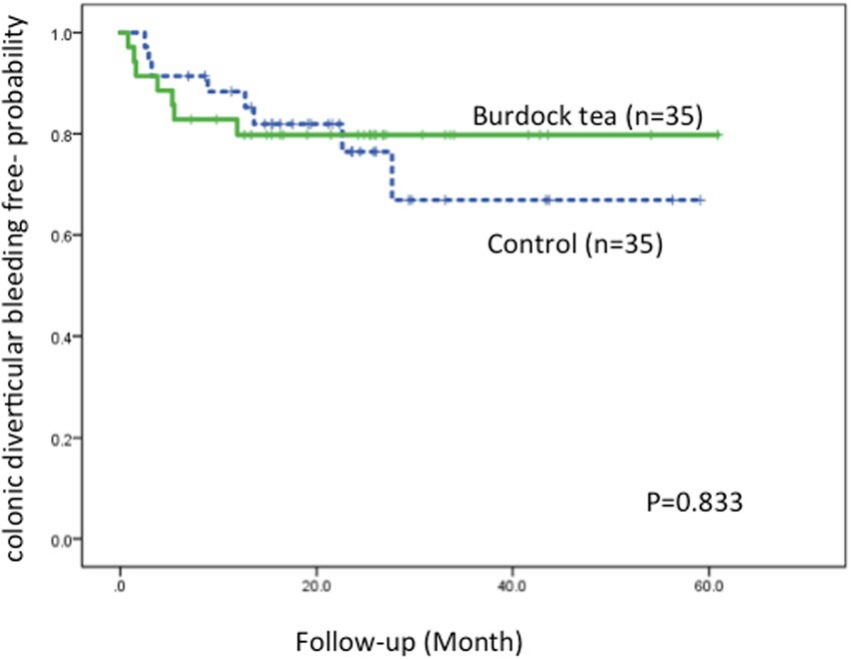
Kaplan-Meier analysis of CDB-free probability. The Kaplan-Meier analysis shows that the CDB- free time is not significantly (p = 0.833 by log rank test) different in the burdock tea and control groups.

The median (interquartile range [IQR]) follow-up period was 16.1 (12.5) months. Seven of 35 (20.0%) patients in the burdock tea group and 8 of 35 (22.9%) patients in the control group experienced recurrence of CDB during follow-up. The rates of recurrence were not significantly different. The Kaplan-Meier analysis showed no significant difference between two groups for CDB-free time (tea group, 49.5 months [95% confidence interval (CI): 41.9–57.1] vs. control group, 44.8 months [95% CI: 36.0–53.6] (Fig. 2).

In study 1, there was no significant difference in age, sex, BMI, history of diverticular bleeding, history of diverticulitis, drinking, smoking, complications, NSAIDs, antiplatelet drugs, laboratory data of Hb, and endoscopic hemostasis between the patients with and without recurrence in the burdock tea-treated group. However, the patients who were treated with antiplatelet drugs or colonoscopy-related medicines showed an increased tendency for recurrence. Of 10 patients with antiplatelet medications, 3 from the burdock tea-treated group and 7 from the control group experienced recurrence twice. Of 8 patients with colonoscopy-related medications, 3 from the burdock tea-treated group had no recurrence, whereas 5 from the control group experienced recurrence twice. There was no significant difference in the rates of CDB recurrence between the patients with and without successful endoscopic therapy.

#### Study 2

**Figure 3:**
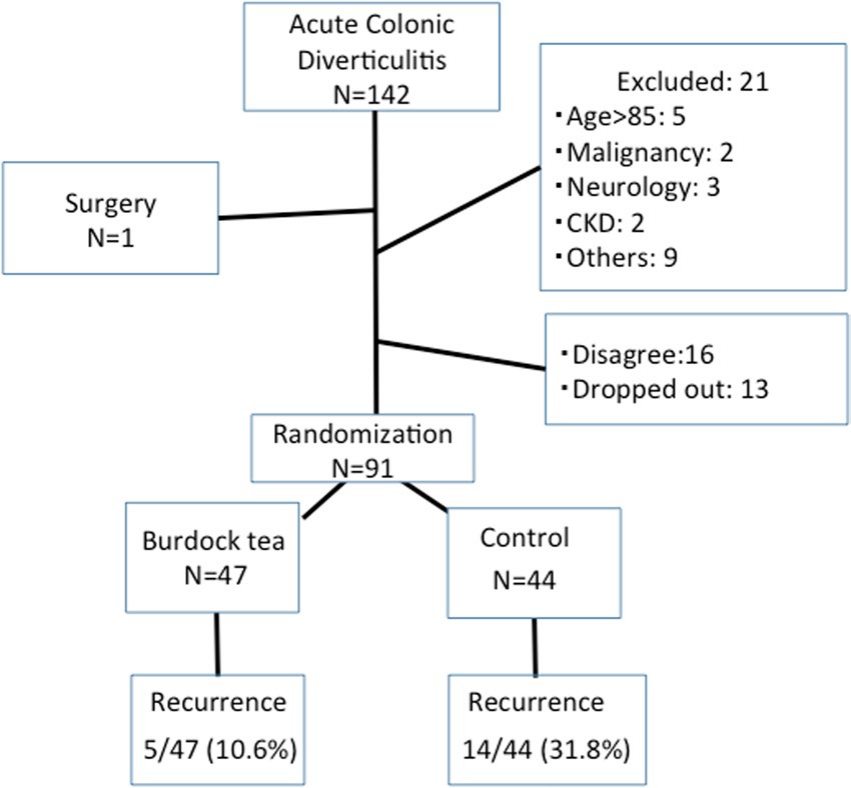
The flow chart of the study population in Study 2.

The median (IQR) follow-up period was 25.6 (16.6) months. The flow chart of participants is shown in Fig. 3.

**Figure 4:**
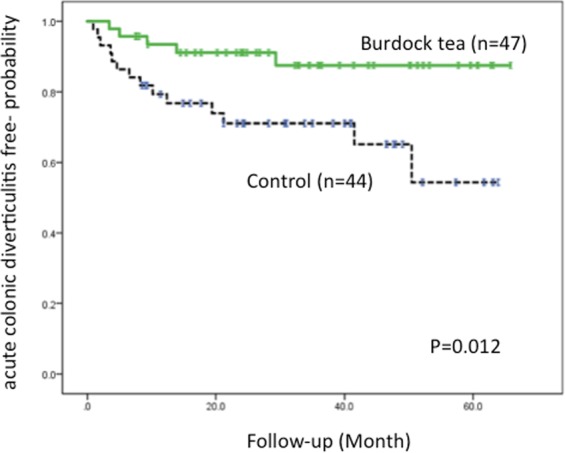
Kaplan-Meier analysis of ACD-free probability. The Kaplan-Meier analysis shows that the ACD-free time in the burdock tea group is significantly (p = 0.012 by log rank test) longer than in the control group.

Five of 47 (10.6%) patients in the burdock tea group and 14 of 44 (31.8%) patients in the control group experienced recurrence of ACD during follow-up. This difference was found to be significant (*p* = 0.013). The Kaplan-Meier analysis showed that the mean ACD-free time in the tea group was significantly longer (p = 0.012 by log rank test) than the control group (59.3 months [95% CI: 54.0–64.7] vs. 45.1 months [95% CI: 37.1–53.0]) (Fig. 4).

In study 2, there was no difference in age, sex, BMI, history of diverticulitis, history of diverticular bleeding, drinking, smoking, complications, and laboratory data of WBC and CRP between the patients with and without recurrence in the burdock tea-treated group.

In both study, there was no any unintended effects except for one mild diarrhea.

## Discussion

In this study, we assessed the efficacy of burdock tea in preventing CDB and ACD recurrences using a randomized, controlled trial (RCT). The results demonstrated that treatment with burdock tea has a possible preventive effect on the recurrence of ACD, but not on the recurrence of CDB.

A recent systematic review of the risk factors for recurrence of ACD showed that the high risk factors were age (young), abscess formation, and history of ACD^36^. Recurrence risk has been reported as being highest in the first year after remission^36^. In this study, the median follow-up period was more than 2 years after remission. Furthermore, although the assignment of patients with a history of ACD was unintentionally larger in the burdock tea group than the control group, burdock tea treatment significantly reduced the rate of recurrence and prolonged the ACD-free time. Thus, we concluded that the burdock tea treatment could be a candidate treatment for prevention against recurrence of ACD.

The effects of the high dietary fiber supplements rifaximin and mesalamine have been discussed in many studies^11–26^. Low dietary fiber intake and constipation are widely accepted as the most important risk factors in asymptomatic diverticulosis^13^. Thus, a high fiber diet and fiber supplements are believed to prevent this condition. Crowe *et al*. reported that consuming a vegetarian diet and high levels of dietary fiber were both associated with a lower risk of admission to hospital or death from diverticular disease^14^. Leahy *et al*. reported that complications and the need for surgery were reduced significantly in patients treated with fiber supplementation^16^. A large cohort study by Strate *et al*.^37^ indicated that prudent dietary pattern decreases the risk of ACD. In the discussion, they mentioned that dietary fiber increases stool bulk, and therefore, decreases the colon pressure and stool transit time. In addition, dietary fiber influences the composition and metabolic activity of the gut microbiota and provides a source of short-chain fatty acids, such as butyrate, which are important for the colon mucosa integrity. A recent review by Rezapour M *et al*.^38^ mentioned that an increased fiber intake may help in reducing the diverticular disease complications. The American Gastroenterology Association (AGA) guidelines on diverticulitis overtly suggest a high dietary fiber intake in patients with a history of acute diverticulitis. In another recent systematic review, the authors recommended the use of liberalized diets over dietary restrictions in adults with acute, uncomplicated diverticulitis. They also strongly recommended a high-fiber diet, which is in accordance with the dietary guidelines, with or without dietary fiber supplementation after an acute episode^39^. On the other hand, Peery *et al*. reported that a high fiber diet and bowel movements were associated with a greater, rather than lower prevalence of diverticulosis^12^. Ornstein *et al*. concluded that dietary fiber supplements (in the commonly used doses) did not relieve constipation^15^. Perhaps the believe that fiber helps prevent diverticular disease is simply a manifestation of Western civilization’s obsession with the need for regular defecation. These findings suggest that the effect of burdock tea on the prevention of ACD recurrence is not solely due to its fiber-rich properties.

Recently, Lee *et al*. reported that burdock roasting tea has high antioxidant properties^40^. In addition, tea is the easiest way to consume health-promoting components from whole foods, containing a combination of multiple polyphenols^41^. The present study showed that all participants demonstrate high compliance (more than 95%), indicating that consumption of burdock tea is possible in the long-term. Thus, burdock tea could actually be a potent strategy for prevention against recurrence of ACD.

We believe that the differences in the effects of burdock tea on preventing CDB and ACD might depend on the different pathogeneses of CBD and ACD^42,43^. Firstly, ACD is a kind of inflammatory disease and burdock tea suppresses the inflammation in the colon diverticulum long-term. The *Arctium lappa* root extracts contain hydroethanolic extracts has the highest phenolic content, and thus, shows the strongest free radical scavenging activity. In addition, iNOS pathway could be associated with the anti-inflammatory effects of arctigenin, a lignan from *A. lappa*^44,45^.

On the other hand, the analysis of risk factors for CDB demonstrated an association with general conditions, such as hypertension and concomitant arteriosclerotic diseases, in addition to the use of nonsteroidal anti-inflammatory drugs^46–48^. Meyers *et al*. showed that diverticulosis does not occur in the presence of inflammation^49^. Thus, the suppression of inflammation by burdock tea may be not associated with the recurrence of CDB. Our results seem to be reasonable considering the differences in the pathogeneses of ACD and CDB.

The treatment with burdock tea was not successful in CDB at least within a year. However, our result showed that there was a tendency for fewer recurrences after 12 months. In general, the recurrence of CDB occurs within two years; thus, we might consider the possibility that burdock tea have a preventive effect on recurrence only after a year of first episode. However, extended studies are needed to prove this possibility. One hypothesis could be that an early recurrence within a year might be caused due to the injured vessels in the same diverticulum and this episode might not be related to an inflammation. In contrast, a late recurrence might be caused due to other diverticula. Burdock tea might be able to modify the formation of subsequent pathogenic diverticulum by its fiber-rich properties.

There were several limitations to the present study that warrant mention. First, this study was performed on Japanese patients and mainly targeted right-sided CDDs. Second, we did not perform a pilot study to determine the optimal dose of burdock tea and sample size. Third, as our study design was not a double masked test, some bias could not be excluded. However, the present study revealed that long-term treatment with burdock tea was feasible and safe. Thus, despite some limitations, long-term treatment with burdock tea seems to be a better therapeutic strategy in preventing recurrence of ACD. Further large-scale, double-blind, dose-response, placebo-controlled, RCT trials are needed to confirm the effectiveness of burdock tea.
